# High-resolution imaging of a cell-attached nanointerface using a gold-nanoparticle two-dimensional sheet

**DOI:** 10.1038/s41598-017-04000-4

**Published:** 2017-06-16

**Authors:** Shihomi Masuda, Yuhki Yanase, Eiji Usukura, Sou Ryuzaki, Pangpang Wang, Koichi Okamoto, Thasaneeya Kuboki, Satoru Kidoaki, Kaoru Tamada

**Affiliations:** 1 Institute for Materials Chemistry and Engineering, Kyushu University, 744 Motooka, Nishi-ku, Fukuoka 819-0395 Japan; 2 Graduate School of Biomedical & Health Science, Hiroshima University, 1-2-3 Kasumi, Minami-ku, Hiroshima City Hiroshima 734-8553 Japan; 3 Graduate School of Science, Nagoya University, Nagoya, 464-8602 Japan; 4 Education Center for Global Leaders in Molecular Systems for Devices, Kyushu University, Fukuoka, 819-0395 Japan

## Abstract

This paper proposes a simple, effective, non-scanning method for the visualization of a cell-attached nanointerface. The method uses localized surface plasmon resonance (LSPR) excited homogeneously on a two-dimensional (2D) self-assembled gold-nanoparticle sheet. The LSPR of the gold-nanoparticle sheet provides high-contrast interfacial images due to the confined light within a region a few tens of nanometers from the particles and the enhancement of fluorescence. Test experiments on rat basophilic leukemia (RBL-2H3) cells with fluorescence-labeled actin filaments revealed high axial and lateral resolution even under a regular epifluorescence microscope, which produced higher quality images than those captured under a total internal reflection fluorescence (TIRF) microscope. This non-scanning-type, high-resolution imaging method will be an effective tool for monitoring interfacial phenomena that exhibit relatively rapid reaction kinetics in various cellular and molecular dynamics.

## Introduction

Fluorescence microscopy has been a key technology in the field of cell biology for the past several decades. Many developments have been achieved related to fluorescent probe technologies for visualizing detailed structures and functions or their dynamic processes in chemically fixed or living cells^1–3^. In most cases, these fluorescence images are collected under the diffraction limit of an optical microscope, e.g., 200 nm in the lateral direction and 500 nm in the axial direction, according to the Abbe and Rayleigh criteria. In this category of fluorescence microscope techniques, confocal laser scanning microscopy (CLSM) has contributed to reaching the theoretical limit and is widely utilized for the imaging of biological samples. The challenge in overcoming the diffraction limit has led to the invention of near-field optical microscopy (SNOM, NSOM) using an evanescent field (non-propagating light) for high-resolution optical imaging^4^. SNOM is designed to monitor the ‘top’ surface of the specimen with a sub-wavelength aperture or a sharpened tip (tip-enhanced or tip-scattering SNOM). Because of this configurational limitation, SNOM is not a standard technique in the fields of biochemistry and cell biology. The most prominent near-field microscopy approach in bio-related fields is total internal reflection fluorescence (TIRF) microscopy^5, 6^. TIRF microscopy enables real-time observation in the 100–200 nm region from the top surface of a cover slip. This technique is suitable for imaging plasma membranes, especially the cell/substrate contact regions where cellular dynamics occur, and processes such as secretory and endocytic processes, the binding of ligands to transmembrane receptors, cytoskeletal dynamics, etc.^7–10^.

Recently, different types of super-resolution fluorescence microscopies have been invented based on completely new concepts, such as stimulated emission depletion (STED) microscopy using patterned illumination and stochastic optical reconstruction microscopy (STORM) with a single-molecule localization approach^2, 11^. The effective point-spread function (PSF) of the three-dimensional (3D) STED microscope has reached sub-diffraction resolution (50 nm × 50 nm × 100 nm) in practical biological samples^12–14^, not only with pulse lasers, but also with gated CW lasers^15^. STORM also enables high resolution, i.e., 20–50 nm for lateral resolution and 100 nm for axial resolution, via combination with TIRF microscopy. These super-resolution fluorescence microscopy techniques represent an advantage in spatial resolution (in particular, lateral resolution) but suffer from a disadvantage in temporal resolution. STORM requires a few seconds to reconstruct a fluorescence image from tens of thousands of frames. Hence, this technique is inadequate for imaging of rapid dynamics in a living system. In addition, these super-resolution fluorescence microscopes remain too expensive and too specific, making them non-standard equipment in basic laboratories.

In this study, we propose a simple, effective method for visualizing the nanointerface of adhesive cells using localized surface plasmon resonance (LSPR) excited on a two-dimensional (2D) self-assembled metallic nanoparticle (NP) sheet^16–18^. The light confined by LSPR detects fluorescence molecules in a region of only a few tens of nanometers at the interface^19, 20^, which creates notably high ‘axially’ confined imaging that is superior to any other super-resolution microscope techniques, including TIRF microscopy. Surface plasmon resonance imaging (SPRI) usually refers to a label-free technique that images refractive index changes at an interface using metal-thin-film-mediated ‘propagating’ SPR^21–23^. The ‘propagating’ SPR has been used for enhanced fluorescence imaging as well^24^. The electric field excited by the propagating SPR is several tens of times stronger than that of the incident light and significantly enhances the fluorescence. However, the disadvantage of these techniques is the spatial resolution caused by their propagating property, more than a few μm at the interface, which reduces the lateral resolution compared with that of a regular optical microscope. There are several ideas to improve the lateral resolution using a specific polarized light, but that would require a scanning system^25^. The penetration depth of the electric field in the axial direction is a few hundred nanometers for the propagating SPR, which is similar to the evanescent field for TIRF imaging. Compared with the existing SPR-microscopes, our LSPR technique exhibits extremely high axial confinement. We also expect an improvement in the lateral resolution because there is little overlap of the excited fluorescence molecules in the depth direction. Currently, no ideal system is available that combines the highest spatial resolution and temporal resolution for biological systems, but we expect that this simple, non-scanning imaging method meets both requirements.

## Results

### Optical field simulation on self-assembled AuOA sheets

**Figure 1:**
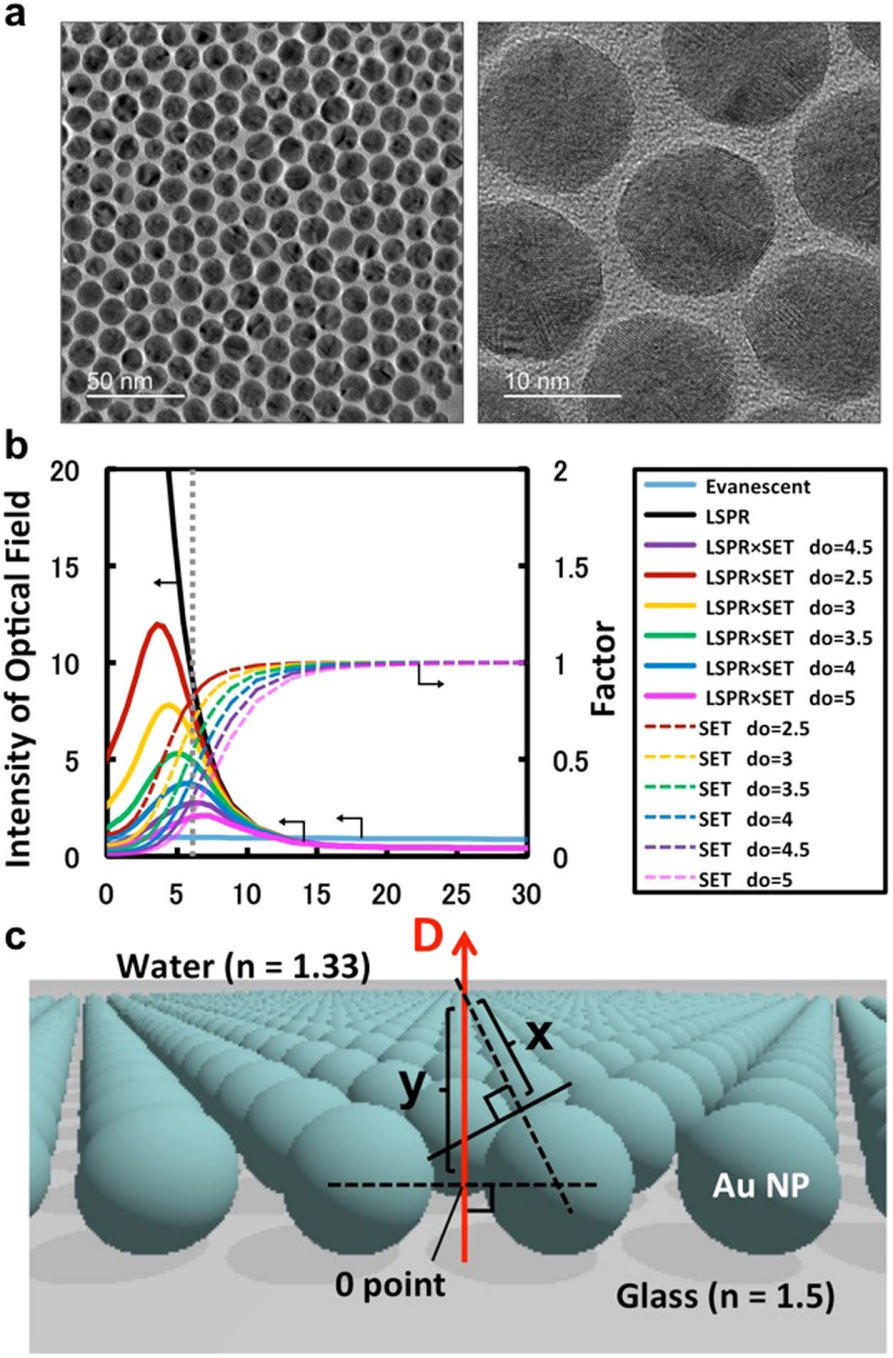
Structure and characteristics of the AuOA sheet. (**a**) TEM images of the AuOA NP sheet; the diameter of the gold core was ca. 12.6 nm and the gap distance was ca. 2.6 nm on average. (**b**) Intensity of the optical field under the influence of LSPR and SET. The black line corresponds to the LSPR field intensity excited on the AuOA sheet calculated by FDTD simulation, and the light blue line corresponds to the evanescent field excited under the TIR condition at the glass/water interface (incident angle: 65°) calculated by Fresnel simulation. The colored dashed lines present the fluorescence attenuation factor due to SET with various SET distances (=*d*_*0*_). The colored solid lines marked as ‘LSPR × SET’ are the LSPR field intensity obtained as the products of LSPR and SET. (**c**) Model of the AuOA sheet for the calculation. The data in (**b**) were obtained on line *D*, where the center position of the adjacent particles was set to *D* = 0. The attenuation factor by SET at each *D* position was calculated based on the distance to the closest particle surface (e.g., at the position of *D* = y, the ‘SET’ value was calculated based on the distance of *d* = x).

Oleylamine-capped gold NPs (AuOA) were used as a component of the self-assembled NP sheets in this study^16^ (see Supplementary Fig. S1). The NP sheets were fabricated at the air-water interface and transferred onto a hydrophobized cover slip (see Supplementary Fig. S2). The diameter of the gold core was ca. 12.6 nm, and the gap distance was ca. 2.6 nm on average. These values were determined by the interdigitated alkyl chains of the oleylamine capping molecules. The self-assembled structure was formed via hydrophobic interactions when the AuOA were spread on the air-water interface (Fig. 1a)^26^. The AuOA in the sheet exhibit a close-packed structure with some defects and domain boundaries on the surface at the nanoscale (smaller than one pixel size). The LSPR band excited on the sheet exhibits a large red-shift (~100 nm) compared with the band in solution, which is due to the collective excitation of the LSPR in the sheet^26^. The macroscopic homogeneity and stability of the 2D films were confirmed via the reproducible Π-A isotherm and UV spectra shown in Supplementary Fig. S2.

The optical field intensity excited by LSPR on the self-assembled AuOA sheet was calculated using the finite-difference time-domain (FDTD) method and is shown in Fig. 1b (black line marked as “LSPR”) based on the model presented in Fig. 1c and Supplementary Fig. S3). In this study, the optical field calculation was performed on line *D*, and the center position of the adjacent particles was set to *D* = 0 (Fig. 1c). The ‘LSPR’ field intensity, which was 50 times stronger at the maximum point (*D* = 1 nm), decayed drastically along the distance of *D* and became less than the original light intensity at *D* = 13 nm. This result indicated that the fluorescence enhancement via LSPR could occur only in the region of *D* < 13 nm in the calculation. The calculated values for the penetration depths of LSPR should be slightly shorter than the actual values in the 2D nanosheet^17^. The enhanced electrical field of the LSPR in the 2D sheet is localized around each nanoparticle, but it can also be transferred to the neighbor particles through plasmon coupling^27^. Thus, the 2D sheet bears the characteristics of enlarged metal structures, and the penetration depth of the 2D sheet must be slightly deeper than that determined by the particle radius. The current calculation with a single pulse excitation does not include the time integral effect, which is necessary to incorporate the effect of the LSPR transfer. Moreover, the periodic boundary model with 3 × 10 particles as the basic unit is too small to see the influence of the LSPR transfer. Based on these reasons, the penetration depth was underestimated in the current FDTD data; however, these calculations are still useful for predicting the nanointerfacial phenomena on the 2D sheet.

The evanescent field excited under the TIR condition at the glass/water interface was calculated via Fresnel simulation and is plotted in Fig. 1b and Supplementary Fig. S3 for comparison (light blue solid line)^5^. The change in the optical intensity depending on the distance *z* is described by equation (4).

4 $${I}_{z}={I}_{0}{e}^{-z/d}$$

In equation (4), *I*_*0*_ is the intensity of the evanescent wave at *z* = 0. The depth of the evanescent wave *d* refers to the distance over which *I*_*0*_ decays to 1/*e*, and *d* is defined by equation (5).

5 $$d=\frac{{\rm{\lambda }}}{4{\rm{\pi }}\sqrt{{{n}_{1}}^{2}{\sin }^{2}{\rm{\theta }}-{{n}_{2}}^{2}}}$$

Additionally, λ is the wavelength of the light, and *n*_*1*_ and *n*_*2*_ are the refractive indices of two media (glass and water, *n*_*1*_ = 1.52 and *n*_*2*_ = 1.33). The decay length of the evanescent wave is significantly longer compared with LSPR, e.g., *d* = 125 nm at *λ* = 561 nm and *θ* = 65°.

Fluorescence quenching due to energy transfer between donor and acceptor molecules is known as Förster resonance energy transfer (FRET). The efficiency of FRET depends on the distance between the donor and the acceptor^28^. The rate of energy transfer in FRET is given by the following equation:

1 $${k}_{FRET}=\frac{1}{{\tau }_{D}}{(\frac{{r}_{0}}{r})}^{6}$$

where *τ*_*D*_ is the lifetime of the donor in the absence of the acceptor, *r* is the distance between the donor and the acceptor, and *r*_*0*_ is known as the FRET distance, i.e., the distance at which the nonradiative energy transfer rate equals the radiative decay rate of the donor in the absence of the acceptor^28–31^.

Energy transfer from a molecular dipole to metal is known to follow another theory, the so-called surface energy transfer (SET), which demonstrates a *d* ^−*4*^ dependence and is described as an analog of FRET:

2 $${k}_{SET}=\frac{1}{{\tau }_{D}}{(\frac{{d}_{0}}{d})}^{4}$$

where *d* is the distance between the excited dipole and the metal surface, and *d*_*0*_ is the SET distance^28, 32–34^. The SET theory predicts the energy transfer independently of the spectral overlap between the dyes and LSPR of the metal NPs, which instead depends on the dielectric constant of the bulk metal^28, 33^. However, as we reported in our previous study, the efficiency of energy transfer between fluorescent dyes and metal NPs certainly depends on the spectrum overlap^35, 36^. Therefore, plasmon-induced resonance energy transfer (PIRET) should be a more accurate explanation of this quenching phenomenon according to the most recent publications^37, 38^. Nevertheless, we considered that the energy transfer between dyes and metal NPs follows the *d* ^−*4*^ dependence given by eq. (2) in this study. The attenuation factor of fluorescence by SET is described as follows:

3 $$\frac{{I}_{r}}{{I}_{\infty }}={[1+{(\frac{{d}_{0}}{d})}^{4}]}^{-1}\,$$

where *I*_*r*_ is the intensity of fluorescence at distance *d*, and *I*_∞_ is the intensity at sufficiently far points, which are plotted by the colored dashed lines (marked as ‘SET’) in Fig. 1b.

The colored solid lines in Fig. 1b marked as ‘LSPR × SET’ indicate the LSPR field intensity obtained as the products of SET and LSPR (the data with the *d* ^−*4*^ dependence are available in Supplementary Fig. S4 for comparison). The ‘LSPR × SET’ value (expected fluorescence enhancement factor) was primarily influenced by the SET distance (*d*_*0*_), although the penetration depth from the interface is not changed by them but is determined by the LSPR profile.

### High-resolution imaging of a cell-attached nanointerface

**Figure 2:**
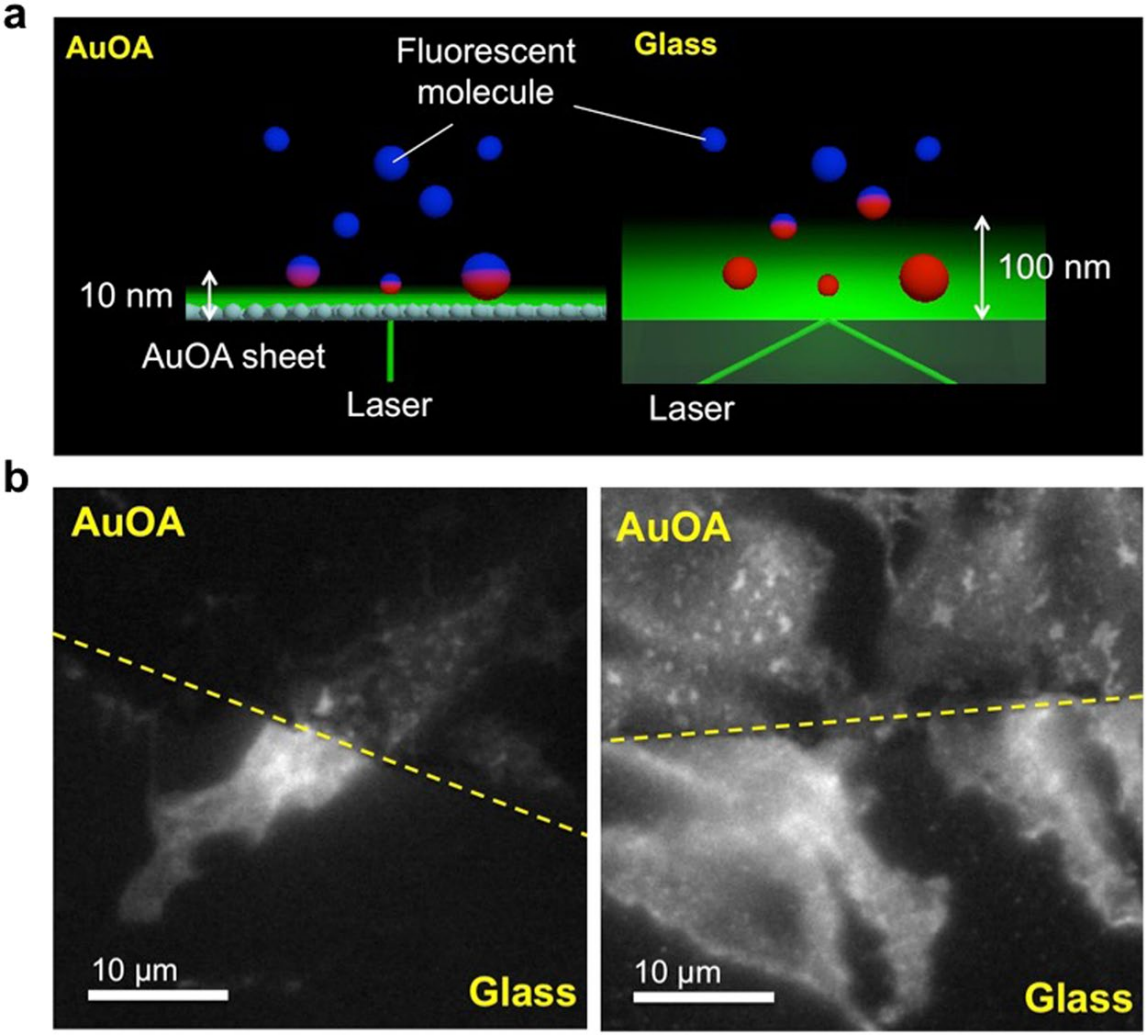
Comparison of interfacial fluorescence images on the AuOA sheet and on glass. (**a**) Schematic drawing of the depth of the LSPR and evanescent fields on the AuOA and glass surface, respectively. (**b)** Fluorescence images of PE-labeled actin filaments in RBL-2H3 cells in aqueous media. The incubation time for the cell adhesion was overnight. The incident light was 561 nm in wavelength, and the intensity was 5 mW. The incident angle was 67° at a critical TIR angle of 61.7°. The exposure time was 30.5 msec (left) or 500 msec (right).

**Figure 3:**
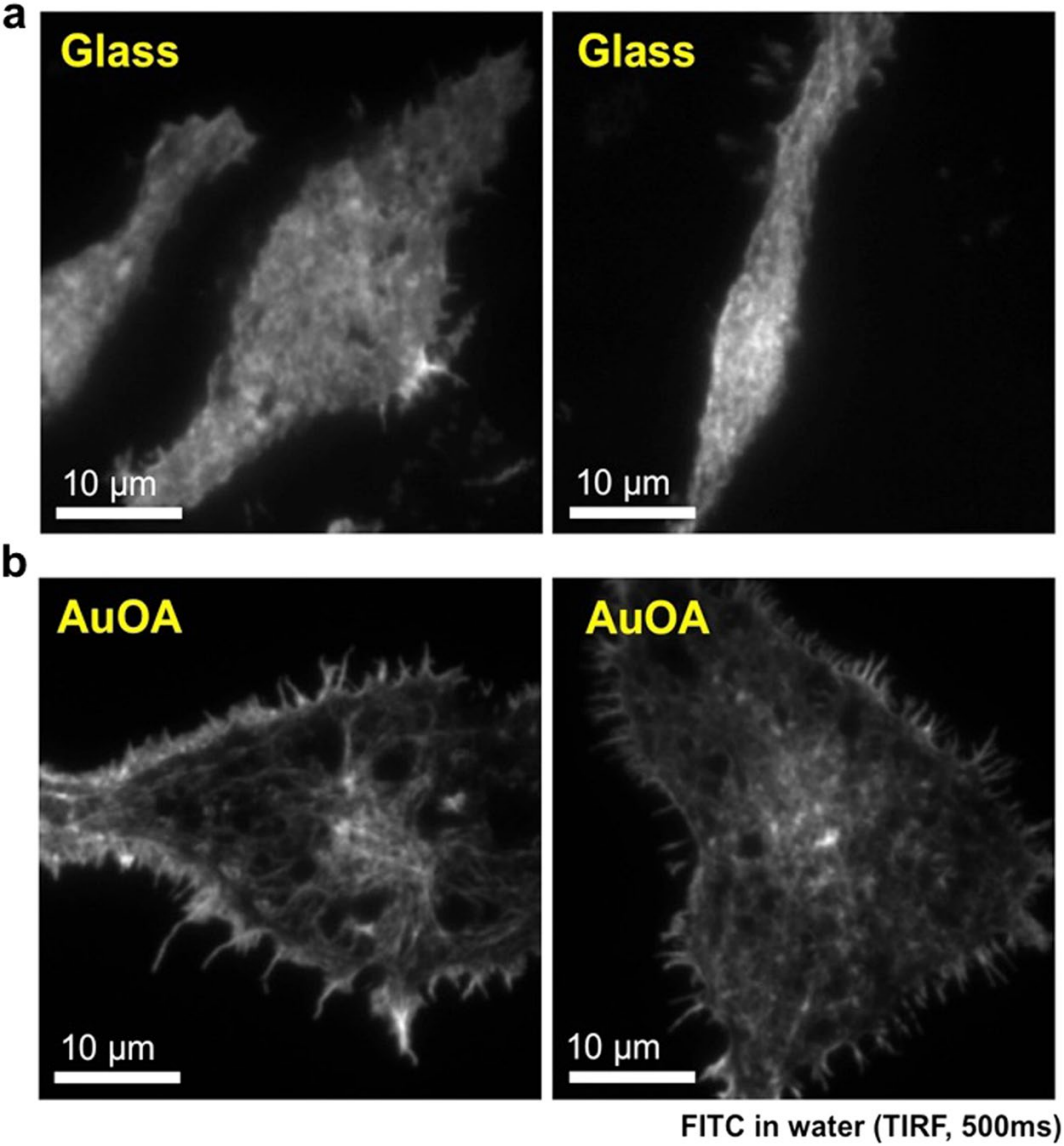
Fluorescence images of FITC-labeled actin filaments in the RBL-2H3 cells. The images were taken on glass (**a**) and on the AuOA sheet (**b**) in aqueous medium. The incubation time for the cell adhesion was overnight. The incident light was 488 nm in wavelength, and the intensity was 5 mW. The incident angle was set at the maximum value (<78° when the critical angle of TIR was 61.4°). The exposure time was 500 msec for all images.

Figure 2a presents a schematic drawing of the confined optical field at the AuOA sheet/water interface and at the TIR interface to show the difference in the visualization depth against fluorescence imaging. Figure 2b shows the fluorescence images of PE-labeled actin filaments in the RBL-2H3 cells in water. In the images, the top half surfaces were covered by the AuOA sheet, and the bottom half surfaces were bare glass. The single cell images taken by crossing the two substrates enabled direct comparison of the images taken on the different substrates (see Supplementary Fig. S5). The effect of confined light due to the AuOA sheet was obvious in the images. Fluorophores close to the AuOA sheets were selectively excited, and the detailed structure of cell-attached interface (focal adhesion) was clearly visualized on the AuOA sheet, whereas these features were screened by the sheet-like actin layer and blurred on glass (regular TIRF image). Although the illumination depth must be shorter on the AuOA sheet than on glass (~1/10 in calculation), the emission intensity was eventually comparable with the aid of LSPR-enhanced fluorescence (see Supplementary Fig. S6). The quality of the image was better after the longer exposure time (500 msec, in Fig. 3b **)**. However, even after the short exposure time (30.5 msec, in Fig. 3a), the nanointerface was successfully recorded.

Figure 3 shows the fluorescence images of the FITC-labeled actin filaments in the RBL-2H3 cells in water. The images were taken at the largest limit of the incident angle (>78° when the critical angle of TIR was 61.4°). Due to the effect of highly inclined light, the signal-to-background ratio increased, and consequently, the spatial resolution was greatly improved for both the TIRF and LSPR images. In particular, the image on AuOA in Fig. 3b revealed the significantly detailed structure of actin filaments at the contact point. The fiber-like brighter areas composed of labeled actin filaments were localized at the edges and center of the cell body, indicating that these components were located in the region of a few 10 nm from the particles (i.e., nano-contacting)^39, 40^. Under close observation, a similar structure could be found on the TIRF images in Fig. 3a as well, although the detail was not as well resolved by the overlap of the emission from the actin layers on top (where the depth of the evanescent wave is estimated to be ca. 65 nm). One reason for the obvious fiber-like structure at the edge of the adhesive cells in Fig. 3b is probably the surface chemical component of the AuOA sheet. The top surface of the AuOA sheet consisted of hydrophobic oleylamine molecules, unlike a glass surface, which might have induced a different cell-attached condition, as shown in Fig. 3b. To avoid this influence, we performed imaging on the AuOA sheet covered with the SiO_2_ sputtered layer as well (The image is shown later).

**Figure 4:**
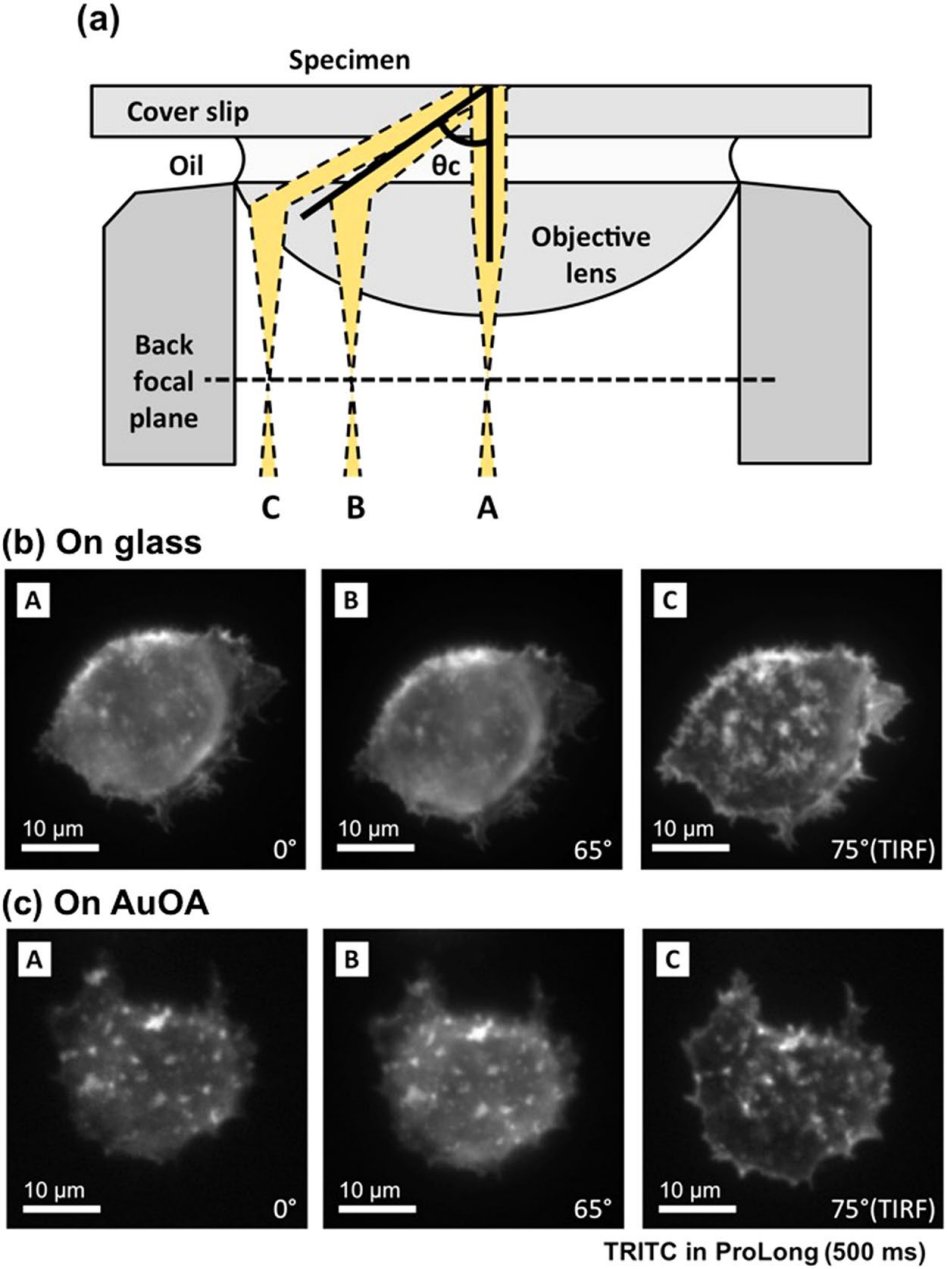
Angular-dependent fluorescence images on glass and on the AuOA sheet. (**a**) Schematic drawing of various incident angles under a TIRF microscope for A: 0°, B: 65°, and C: 75° when the critical angle of TIR *(θ*_*c*_) was 74.4°. (**b**) Fluorescence images of the TRITC-labeled actin filaments in the RBL-2H3 cells in aqueous media. The images were taken with a laser 561 nm in wavelength (5 mW), and the exposure time was 500 msec. The incubation time for the cell adhesion was 30 min, when the cells formed round shapes indicating an early stage of adhesion (see Supplementary Fig. S7).

The high potential of this LSPR-imaging technique was demonstrated by the experiments conducted under different illumination angles, as shown in Fig. 4. We illuminated the specimen with three different incident angles: (A) normal incidence, *θ* = 0°, (B) oblique incidence, *θ* = 65° (<*θ*_*c*_), and (C) TIR incidence, *θ* = 75° (>*θ*_*c*_). The cells were labeled with TRITC dyes and mounted in ProLong (n = 1.46). ProLong is a glycerol-based liquid mounting medium applied directly to fluorescence-labeled cells on microscope slides to protect the dyes from photofading and to cure the samples for longer-term storage. The critical angle of TIR at the glass and ProLong interfaces was 74.4°. Figure 4b show images taken on a glass substrate. As expected, the only TIRF image (**C**) visualized the detailed structure of the cell-contacting area, in stark contrast with the regular epifluorescence (**A**) and oblique illumination fluorescence (**B**) images. However, the bright spots and fine structures on the adhesive region were resolved with high sensitivity at all irradiation angles when the AuOA sheets were used as imaging substrates.

**Figure 5:**
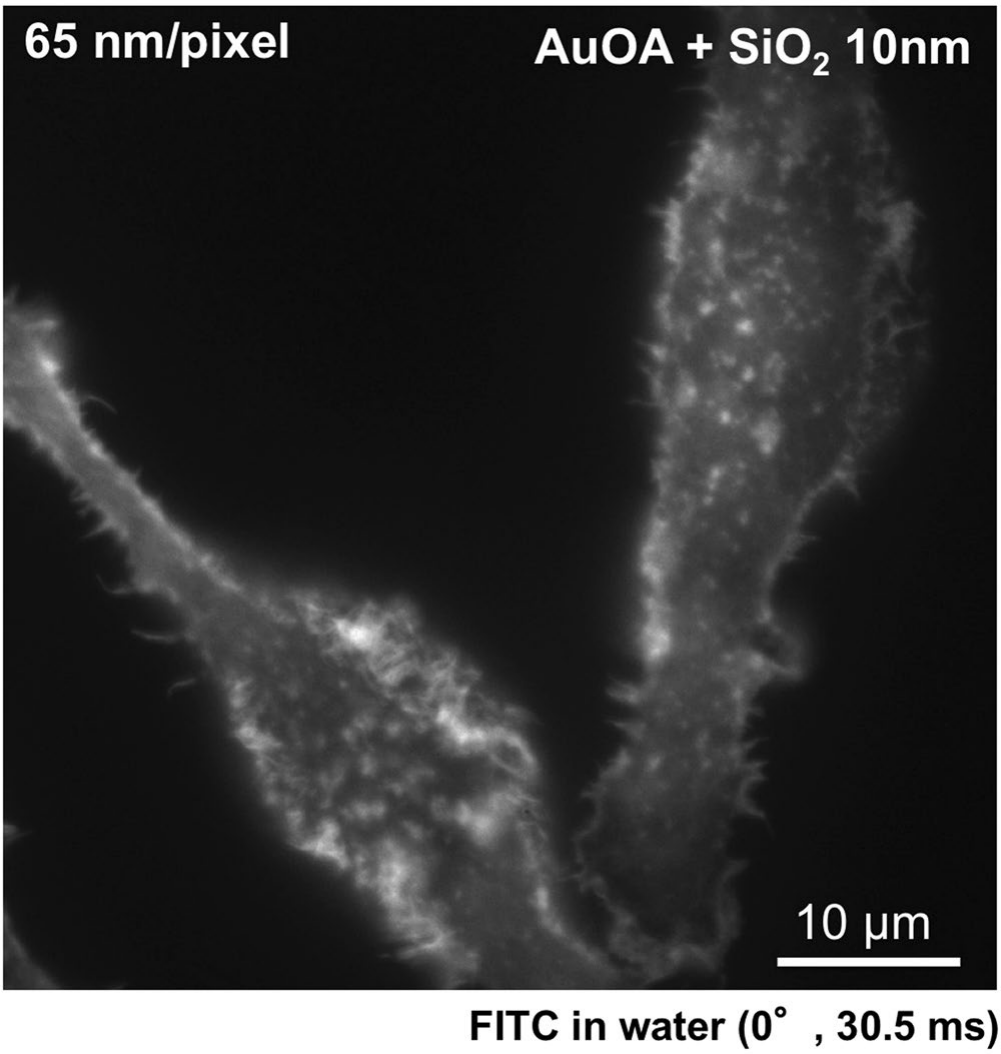
High spatiotemporal resolution image of the FITC-labeled actin filaments in RBL-2H3 on the SiO_2_-AuOA sheet. The image was taken under an epifluorescence microscope (incident angle: 0°) in aqueous medium with a super-resolution digital CMOS camera (65 nm/pixel). The cells were attached on the AuOA sheet with a 10 nm thick SiO_2_ layer. The images were taken with a laser 561 nm in wavelength (5 mW), and the exposure time was 30.5 msec. The incubation time for the cell adhesion was the same as that in Fig. 3 (overnight). The image taken by a regular TIRF camera (160 nm/pixel) is available in Supplementary Fig. S9 for comparison. The magnified images are available in Supplementary Fig. S10.

The last challenge was to determine how high spatial-temporal resolution images could be captured under an epifluorescence microscope. For this purpose, we used FITC-labeled cells, which produced most of the fine structural images in the previous attempt (Fig. 3a). Figure 5 shows the image taken under an epifluorescence microscope for the same FITC-labeled cells on the AuOA sheet covered with 10 nm SiO_2_ layer. Although the experiment of fluorescence bead confirmed that 20 nm SiO_2_ layer provided the maximum fluorescence enhancement (Supplementary Fig. S8), we chose the 10 nm SiO_2_ layer in consideration of the thickness of extracellular matrix^41^. The exposure time was 30.5 msec. We obtained the fluorescence image of cells that show elongated morphology. The morphology is identical to that observed on glass (Fig. 3a) but is highly sensitive to the cell-contacting nanointerfacial structure. Because the lateral resolution of the original image appeared to exceed the TIRF camera resolution (160 nm/pixel), we switched to a super-resolution digital CMOS camera (ORCA-Flash 4.0, Hamamatsu, Japan), which delivers 65 nm/pixel lateral resolution in combination with a 100 × objective lens and captured the image shown in Fig. 5. The image taken using the 160 nm/pixel lateral resolution camera is presented in Supplementary Fig. S9, and the comparison between the magnified images is shown in Supplementary Fig. S10. Our success in obtaining a high spatiotemporal resolution image without a scanning operation in this study demonstrates the potential of this LSPR-assisted microscope technique for live-cell imaging and monitoring nanointerfacial phenomena, including relatively rapid cellular or molecular dynamics (the test experiment for the live-cell imaging is available in the Supplementary Videos S1–S3)^42–44^.

## Discussion

The obtained structures (spot-like or fiber-like) cannot be perfectly explained, but they may be due to the influence of the labeling reagents and their protocols. Nevertheless, our results were clearly consistent with the most recent morphological study of actin cytoskeleton with dual-objective STORM. Xu *et al*. succeeded in obtaining a super-resolution image of the actin network with STORM, i.e., <10 nm lateral resolution and <20 nm axial resolution^40^. The spatially resolved individual actin filaments in their cells revealed the existence of ‘ventral’ and ‘dorsal’ actin layers. The morphology of the ‘ventral’ layer that they reported was similar to that of our TIRF image (e.g., Figs 3b or 4b). Furthermore, the color-coded z-positions revealed that the lowest z-positioned area (i.e., the area closest to the substrate) was located at the edge of the cell (referred to as the ‘cell protrusion’) and the bottom of the cell, with isolated spot-like structures that must correspond to focal adhesions at the end of the actin bundles^45–47^. Unfortunately, the absolute z-position was not mentioned in their paper. However, we were confident of our result due to the morphological agreement with the state-of-art STORM data^47^. Because none of the other techniques are currently able to achieve a few tens of nm confinement, we plan to continue to gather the necessary information on the absolute z-position, probably with the use of a fluorescence marker with nano-distance control, to add the scale of the z-axis to our nanointerfacial images.

Notably, the light confined by LSPR improved not only the ‘axial’ but also the ‘lateral’ resolution in the practical images. The combination of the light confinement at the nanointerface (non-propagating LSPR) and the enhanced fluorescence is the key to realizing notably a high signal-to-background ratio and high contrast (the quantitative data from using the fluorescence beads are available in Supplementary Fig. S10). In the current system, the broad extinction spectra of AuOA overlapped with the excitation/emission spectra of the chosen dyes (FITC, PE and TRITC) and is considered a contributor to the fluorescence enhancement (see Supplementary Fig. S11).

In conclusion, we have demonstrated the high axial confinement and improved lateral resolution fluorescence imaging of a cell-attached nanointerface with labeled actin filaments using a self-assembled gold-NP sheet (AuOA) as the imaging substrate. The simple idea of using homogeneously excited LSPR as the imaging plate added new value to conventional epifluorescence microscopy and can deliver high-quality fluorescence images comparable to or better than those of TIRF microscopy in certain cases. This non-scanning, high-resolution imaging method is expected to become an effective tool for monitoring interfacial phenomena that exhibit relatively rapid reaction kinetics in various cellular and molecular dynamics.

## Methods

### Synthesis of gold NPs

Oleylamine-capped gold NPs (AuOA) were synthesized using the method reported by Hiramatsu *et al*.(Supplementary Fig. S1)^48^ First, 411 mg (1.0 mmol) of gold (III) chloride acid 4-hydrate and 5 mL (15.2 mmol) of oleylamine were dissolved in 50 mL of toluene and heated to 100 °C for 60 min. The reaction solution was held at 90 °C for another 180 min. When the solution was cooled to room temperature, the AuOA NPs were extracted by ultracentrifuge and purified several times to remove excess oleylamine. The purified AuOA NPs were redispersed in toluene.

### Fabrication of self-assembled monolayers composed of AuOA

The AuOA NP 2D self-assembled monolayer was fabricated at the air-water interface in an LB trough (KSV NIMA, Sweden), as described in our previous study (Supplementary Fig. S2)^16^. The AuOA NP dispersion in toluene was spread on water. After toluene evaporated, solid-like domains were formed on the water surface by self-assembly of the AuOA NPs. The solid-like domains were gathered using a Teflon bar and compressed until the surface pressure reached 15 mN/m. The sheet was transferred onto a cover slip hydrophobized by hexamethyldisilazane (HMDS) via the Langmuir-Schaefer (LS) method^16^. Both silver and gold NPs can excite LSPR under visible light, and silver is known to exhibit a stronger electric field than gold. However, because silver exhibits cytotoxicity, unlike gold, we used gold NPs in this study. In fact, we could obtain better adhesion of cells on the AuOA sheet than on the Ag NP sheet (myristate-capped Ag NPs), which was identical to that on glass (Supplementary Fig. S5). The electron microscopy (TEM) images of AuOA sheets were captured using a JEM-ARM200F instrument (JEOL, Tokyo, Japan). The AuOA sheet was prepared on the pristine surface of a TEM grid (U1015: EM Japan) by the LS technique and observed with an acceleration voltage of 200 kV.

### Preparation of biological samples

Rat basophilic leukemia (RBL-2H3) cells were cultured in Roswell Park Memorial Institute (RPMI) 1640 medium supplemented with 10% fetal calf serum (FCS), 100 U/mL penicillin, and 100 μg/mL streptomycin^49^. The day before the experiments, RBL-2H3 cells were harvested using trypsin. A flexiPERM® chamber conA (φ12 mm, Greiner Bio One) was placed on the glass slip half-covered with the AuOA NP sheet to make a ‘well’ to retain the cell solution. The AuOA substrates were sterilized by UV irradiation for 30 min prior to use. RBL-2H3 cells in RPMI 1640 medium were placed into the well and cultured for the indicated time (30 min or overnight) in a CO_2_ incubator. The cells were immobilized using 4% paraformaldehyde for 10 min and disrupted using 0.1% Triton X-100 in a phosphate buffered saline (PBS) solution for 10 min. After rinsing twice with PBS buffer, actin filaments were separately stained with fluorescent dye, namely, TRITC (Sigma-Aldrich), FITC (Thermo Fisher), or PE (Sigma-Aldrich). In the case of TRITC and FITC, the TRITC or FITC conjugates of phalloidin were used to label the actin filaments. In the case of PE, the actin filaments were stained using indirect staining protocols (two-step reactions). First, the biotin-phalloidin (Thermo Fisher) was bound to the actin filaments, and streptavidin-PE (BD Pharmingen, USA) was immobilized by avidin-biotin interaction. The cell imaging was conducted in water or in ProLong antifade reagent (Thermo Fisher, USA) as a mounting medium. For live-cell imaging, the 3T3 fibroblasts with stably expressed venus-paxillin were cultured in Dulbecco’s modified Eagles medium (DMEM)-low glucose medium supplemented with 10% fetal bovine serum (FBS), 100 U/mL penicillin, 100 μg/mL streptomycin and 250 μg/mL G418^50, 51^. The AuOA sheet was rinsed twice with sterile PBS, replaced with the culture medium and pre-incubated at 37 °C for 30 min. The cells were trypsinized, seeded onto the sheet and maintained in the CO_2_ incubator overnight. Prior to the live cell imaging, the culture medium was replaced with phenol red free CO_2_ independence Leibovitz’s (L-15) medium, which was supplemented with 10% FBS and penicillin/streptomycin. The imaging was performed in a humidified temperature controlled chamber at 37 °C.

### TIRF microscopy

The fluorescence images were captured using a customized TIRF microscope (ECLIPSE Ti, Nikon, Japan) equipped with a multiple-wavelength laser (LightHUB, Omicron-Laserage, Laserprodukte GmbH, Germany), a high-speed CCD camera (EM-CCD C9100-13, Hamamatsu Photonics, Japan) and a 100 × objective lens (CFI Apo TIRF 100 × H/1.49, Nikon, Japan). The multiple-wavelength laser was used to choose the optimum incident wavelength in consideration of the overlap with the LSPR wavelength of the AuOA sheet and the excitation/emission wavelengths of the fluorescent dyes. In this study, 488 and 561 nm lasers and 525 and 609 nm emission filters were applied for the three dyes (see Supplementary Fig. S11). The incidence angle was adjusted from perpendicular to beyond the total internal reflection angles. The quality of the fluorescence images captured on the AuOA sheet was compared with the quality of images taken on glass under the same conditions, e.g., incident light intensity and wavelength, incident angle, fluorescence filter and exposure time. All images were collected without an aperture stop (AS) and shown without contrast control via graphics processing software.

### FDTD calculation

The intensity of the optical field excited on the AuOA sheet was calculated by the FDTD method using the commercial software Poynting for Optics (FUJITSU, Japan). In a manner similar to that described in our previous study^17, 18^, the dielectric function of Au was approximated by the Drude formula based on the literature values reported by Johnson and Christy. A periodic boundary condition was set up in the X and Y directions with a basic unit composed of 3 × 10 particles. A perfectly matched layer-absorbing boundary condition was set in the Z direction. A pulsed light composed of a differential Gaussian function with a pulse width of 0.5 fs was used as an excitation source. A non-uniform mesh was used with a grid size of 0.3 nm to 5 nm.

## Electronic supplementary material

Supplementary information

Supplementary Video S1(A)

Supplementary Video S1(B)

Supplementary Video S2(A)

Supplementary Video S2(B)

Supplementary Video S3(A)

Supplementary Video S3(B)
