# Supplementary material for: High-resolution imaging of a cell-attached nanointerface using a gold-nanoparticle two-dimensional sheet
Source: Sci Rep. 2017 Jun 16;7:3720. doi: 10.1038/s41598-017-04000-4 (PMC5473937; doi:10.1038/s41598-017-04000-4)
Supplement: Supplementary file 1 — Supplementary information [file 41598_2017_4000_MOESM1_ESM.pdf]

Supplementary information

High-resolution imaging of a cell-attached nanointerface using a gold-nanoparticle two-dimensional sheet

Shihomi Masuda,[1] Yuhki Yanase,[2] Eiji Usukura,[3] Sou Ryuzaki,[1] Pangpang Wang,[4] Koichi Okamoto,[1] Thasaneeya Kuboki, [1] Satoru Kidoaki, [1] Kaoru Tamada[1]

[1] *Institute for Materials Chemistry and Engineering, Kyushu University, 744 Motooka, Nishi-ku, Fukuoka, 819-0395, Japan.*

[2] *Graduate School of Biomedical & Health Science, Hiroshima University, 1-2-3 Kasumi, Minami-ku, Hiroshima City Hiroshima, Japan 734-8553, Japan*

[3] *Graduate School of Science, Nagoya University, Nagoya, 464-8602, Japan*

[4] *Education Center for Global Leaders in Molecular Systems for Devices, Kyushu University, Fukuoka, 819-0395, Japan.*

Correspondence and requests for materials should be addressed to K.T. (tamada@ms.ifoc.kyushu-u.ac.jp)

Table of contents:

Supplementary Fig. S1: Schematic illustration of the synthesis route for AuOA
Supplementary Fig. S2: Schematic illustration of the fabrication of the AuOA sheet
Supplementary Fig. S3: Intensity of the optical field excited by LSPR and evanescent light.
Supplementary Fig. S4: Comparison of FRET and SET efficiencies
Supplementary Fig. S5: Optical microscope images of cells attached on glass and the AuOA sheet
Supplementary Fig. S6: Quantitative image analysis of Fig. 2b
Supplementary Fig. S7: Fluorescence images of TRITC-labeled RBL-2H3 cells on the AuOA sheet at different incubation times
Supplementary Fig. S8: Snapshot of single fluorescence beads
Supplementary Fig. S9: Fluorescence image of FITC-labeled RBL-2H3 on SiO_2-AuOA sheet captured with a regular TIRF-CCD camera
Supplementary Fig. S10: Magnified images of Fig. 5 and Supplementary Fig. S9
Supplementary Fig. S11: Spectrum overlap between LSPR band of the AuOA sheet and fluorescence spectra of fluorescent dyes
Supplementary Video S1: Live cell imaging of Venus-paxillin-expressing NIH-3T3 cells on an AuOA sheet.
Supplementary Video S2: Live cell imaging of Venus-paxillin-expressing NIH-3T3 cells on glass.
Supplementary Video S3: Time-lapse imaging of Venus-paxillin-expressing NIH-3T3 cells on an AuOA sheet.

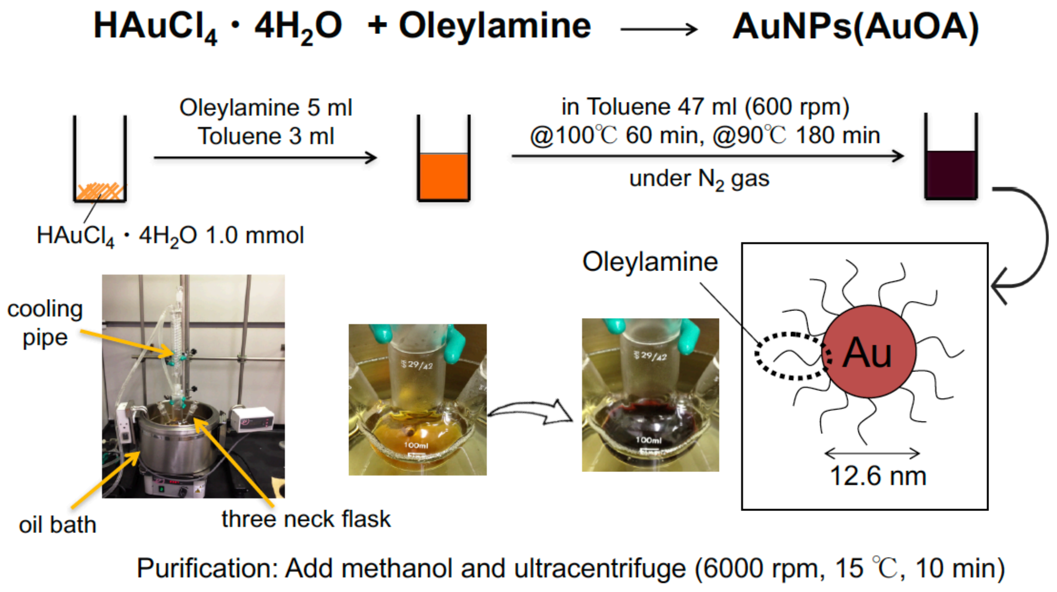

Supplementary Fig. S1: Schematic illustration of the synthesis route for AuOA. An amount of 411 mg (1.0 mmol) of gold (III) chloride acid 4-hydrate and 5 mL (15.2 mmol) of oleylamine were dissolved in 50 mL of toluene and heated to 100 °C for 60 min. The reaction solution was held at 90 °C for another 180 min. When the solution was cooled to room temperature, the AuOA were extracted by ultracentrifuge and purified several times to remove excess oleylamine. The purified AuOA were redispersed in toluene.

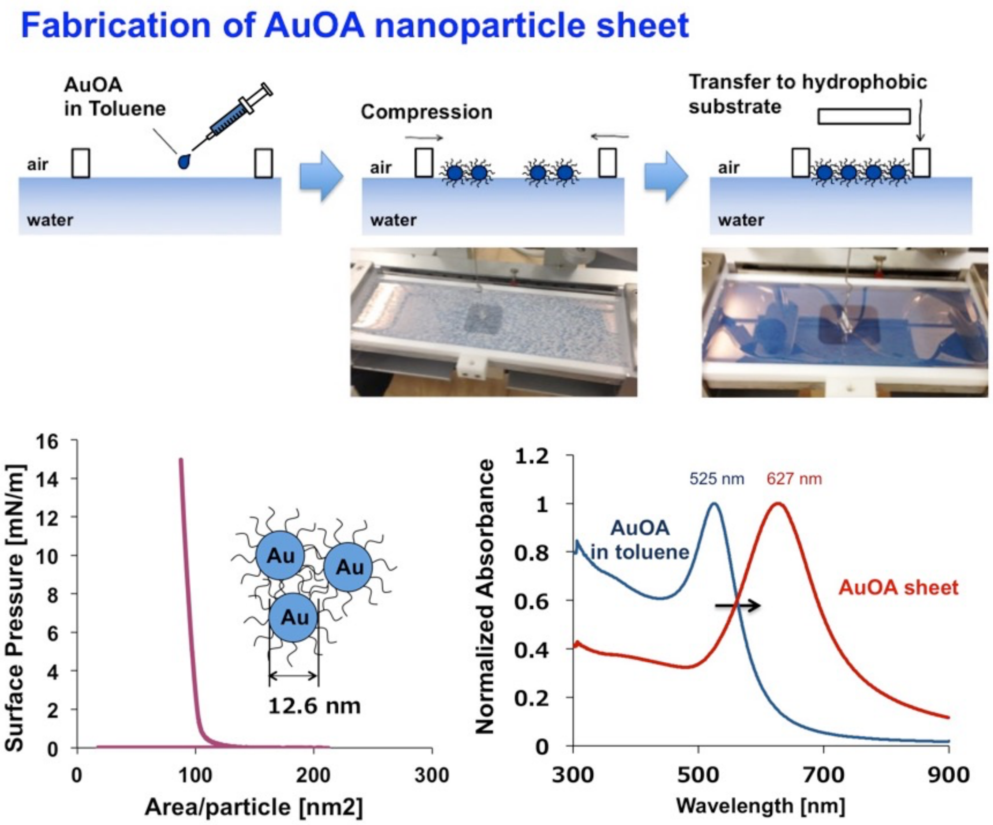

Supplementary Fig. S2: Fabrication procedure for self-assembled monolayers composed of AuOA. The AuOA dispersion in toluene was spread at the air-water interface in an LB trough. After evaporation of toluene, solid-like domains were formed on water surface by self-assembly of AuOA NPs. The solid-like domains were gathered using a Teflon bar and compressed until the surface pressure reached 15 mN/m. The sheet was transferred onto a cover slip hydrophobized by hexamethyldisilazane (HMDS) via the Langmuir-Schaefer (LS) method. The bottom figures show the surface pressure (Π) - area per particle (A) curve (left) and the absorption spectra (right, dispersion in toluene (blue) and self-assembled monolayer sheet (red)). The large red-shift of the LSPR band by the sheet formation originates from the long-range interaction of LSPR in the sheet, as described in our previous studies[1,2].

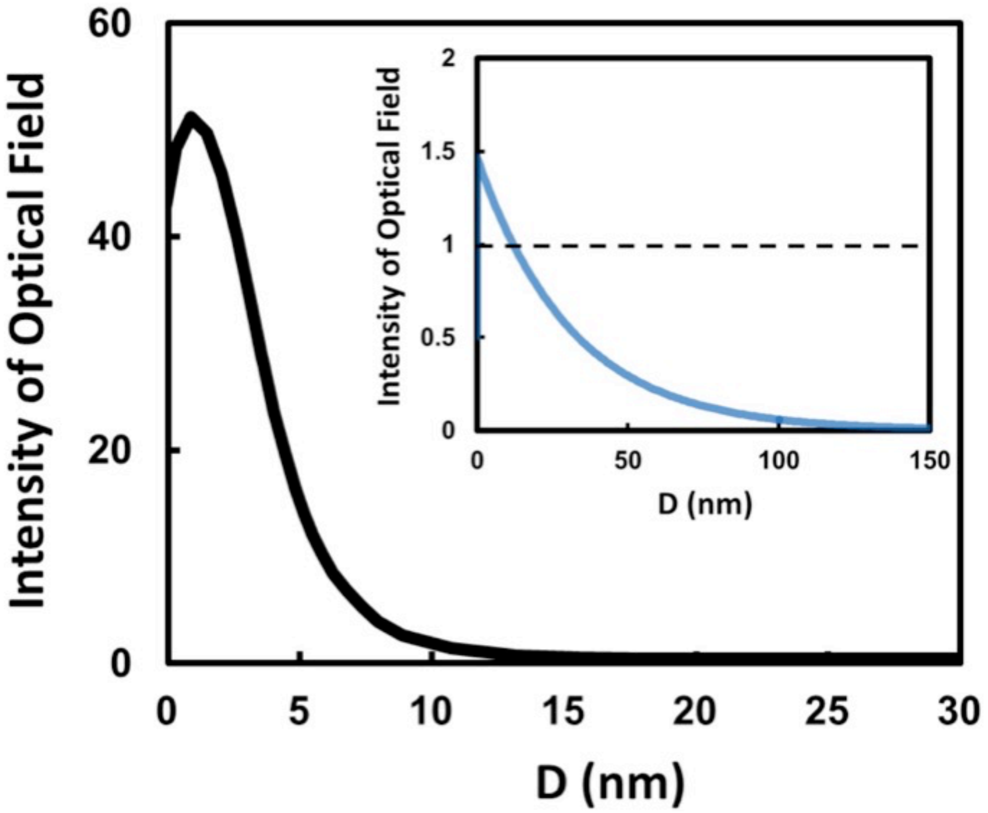

Supplementary Fig. S3: Intensity of the optical field excited by LSPR and evanescent waves. The LSPR field intensity excited on the AuOA sheet was calculated using the finite-difference time-domain (FDTD) method based on the model presented in **Figure 1c**. The inset shows the evanescent field excited at the glass/water interface under the TIR condition calculated by Fresnel simulation. The penetration depth of LSPR is much shorter (~13 nm) than that of the evanescent wave (~150 nm).

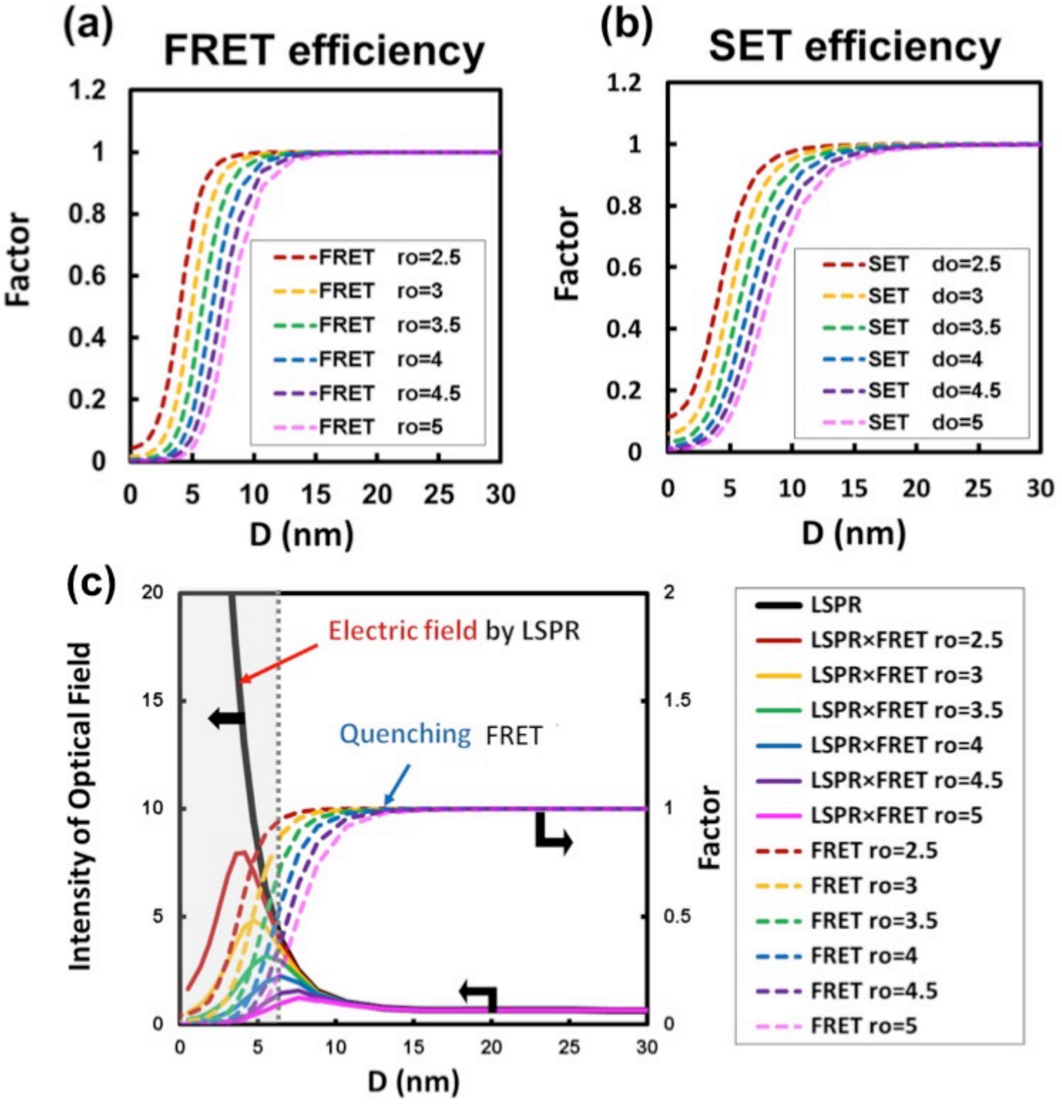

Supplementary Fig. S4: Comparison of the FRET and SET efficiencies. (a) FRET and **(b)** SET profiles calculated via eqs. (1) and (2). The colored dashed lines present the fluorescence attenuation factor due to FRET and SET with various FRET (= r_0) and SET (= d_0) distances. The profiles of FRET and SET are slightly different; the FRET curve is steeper than the SET curve due to the r^{-6} dependence instead of d^{-4} dependence. **(c)** Intensity of the optical field under the influence of LSPR and FRET, for comparison with the SET system shown in **Figure 1b.** The colored solid lines marked as 'LSPR×FRET' correspond to the products of LSPR and FRET, which are slightly weaker than that of LSPR and SET.

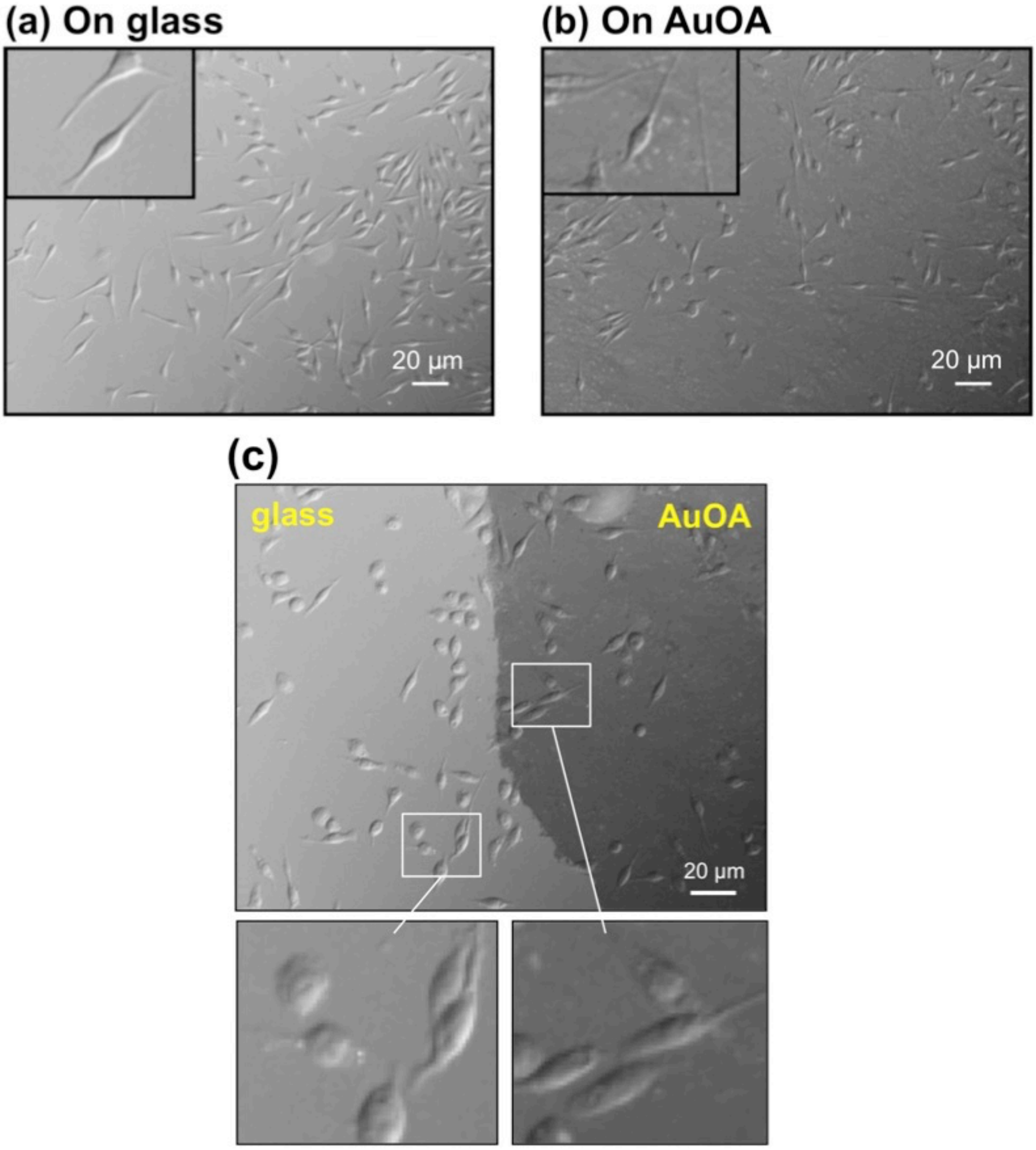

Supplementary Fig. S5: Optical microscope images of cells attached on glass and the AuOA sheet. **(a)** RBL-2H3 cells attached on glass slip and **(b)** on the AuOA sheet. These cells were cultured independently in isolated chambers overnight. **(c)** RBL-2H3 cells cultured on a glass slip half covered with AuOA sheet in the same chamber. This optical microscope image was taken after immobilization and staining by TRICT dye.

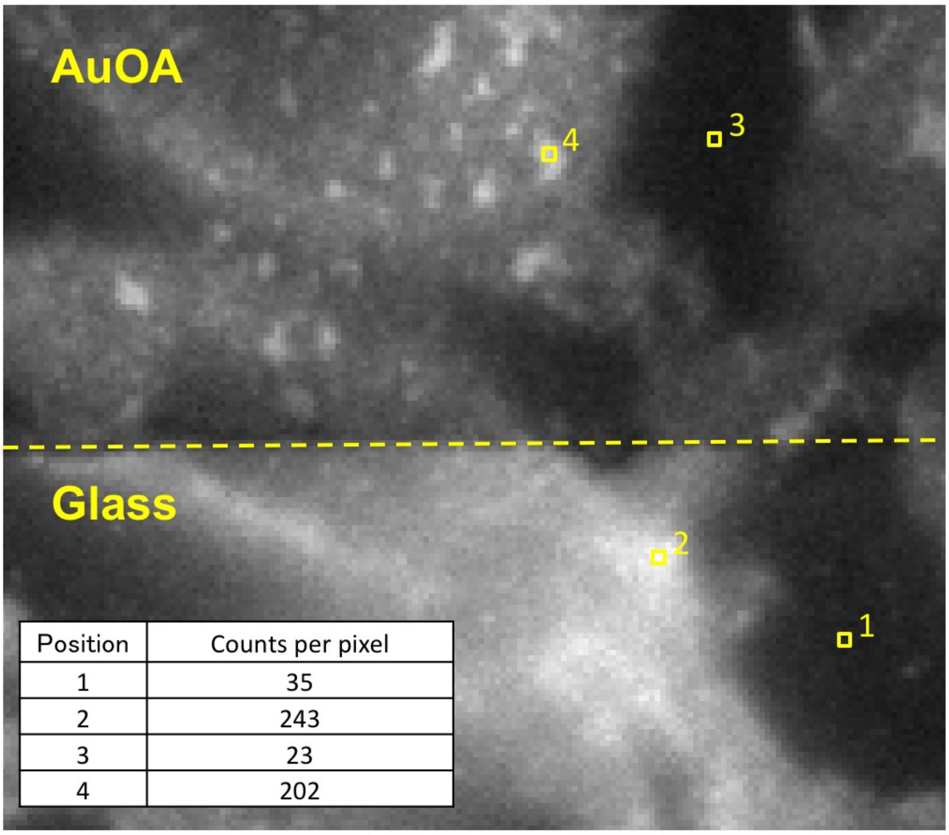

Position	Counts per pixel
1	35
2	243
3	23
4	202

Supplementary Fig. S6: Quantitative image analysis of Fig. 2b. Comparison of the contrast in the images on AuOA and on glass in Fig. 2b (right). The dark and bright areas composed of 4 pixels were selected on each image, and the averaged counts per pixels are summarized (pixel size: 160 nm). Despite the different imaging depths, both surfaces exhibited comparable contrasts.

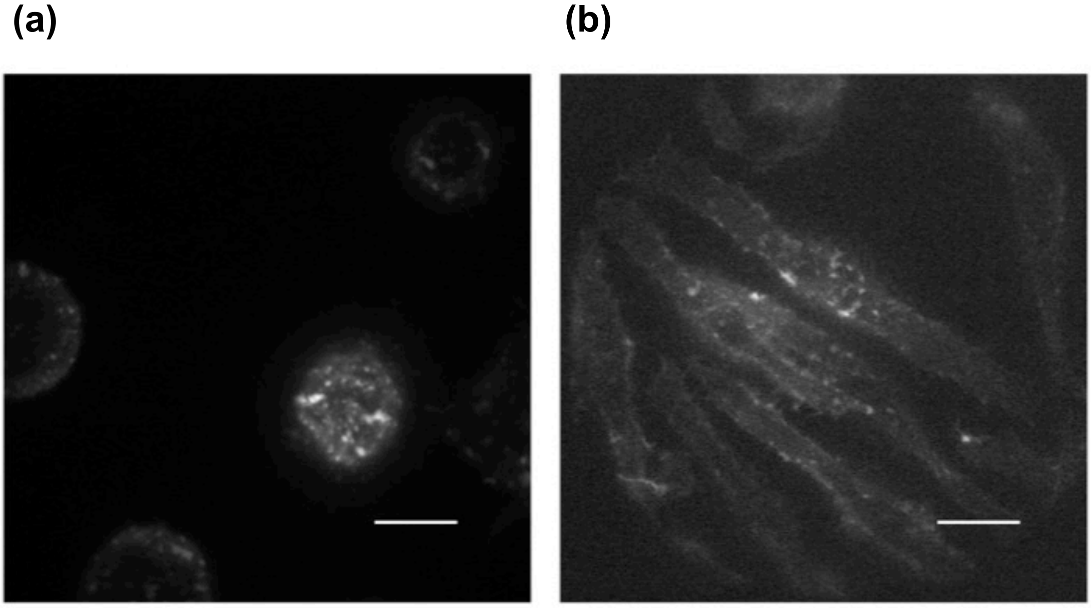

Supplementary Fig. S7: Fluorescence images of TRITC-labeled RBL-2H3 cells on the AuOA sheet at different incubation times. (a) Incubation time of 30 min and **(b)** overnight. The images were taken in aqueous medium with a laser with a wavelength of 561 nm (5 mW) and a fluorescence filter of 609 nm. The incident angle was 0°, and the exposure time was 500 msec. The scale bar is 10 μm. As the incubation time increased, the cells exhibited elongated shapes. However, the number of spots for focal adhesion did not particularly increase.

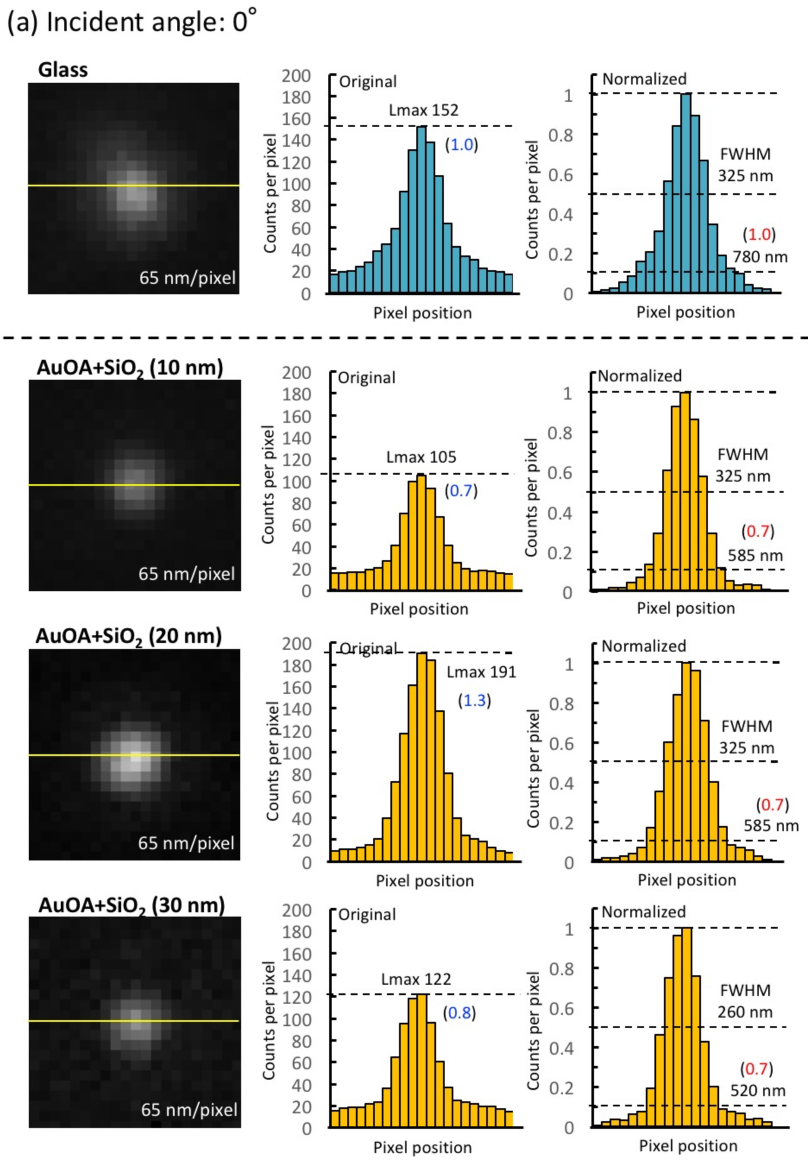

Supplementary Fig. S8(a): Snapshot of single fluorescence beads (incident angle: 0°). The images of fluorescence beads on the AuOA sheet were compared with that on glass. The fluorescence beads utilized were FluoSpheres (carboxylate-modified microspheres, Ex540/Em560, 200 nm in diameter), which were spontaneously adsorbed on the substrate in water. The fluorescence intensity of the beads was slightly weaker on AuOA sheet covered with 10 nm and 30 nm SiO_2 layers, while it was stronger with 20 nm SiO_2 layer. Although the illumination depth was much shorter on the AuOA sheet (a few 10 nm in calculation), the emission intensity was comparable with that on glass due to the LSPR-enhanced fluorescence. Although the FWHM values were the same for all of the images, the foot section of the profile presented differences between the images taken on glass and the AuOA sheet. The width at 10% of the maximum intensity was 12 pixels (780 nm) on glass and 8-9 pixels (520-585 nm) on the AuOA sheet. The wavelength of the incident laser was 514 nm, and the intensity was 1 mW. The exposure time was 100 msec for all images.

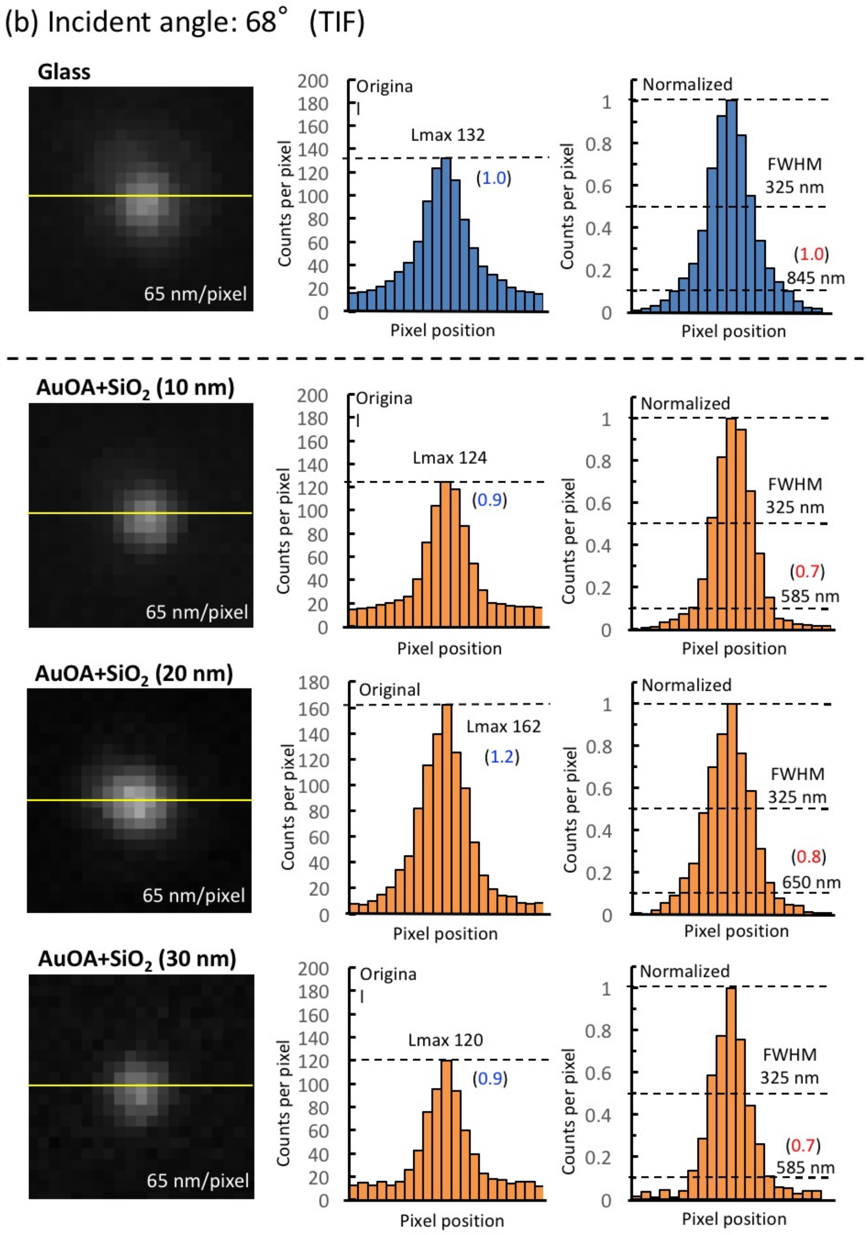

Supplementary Fig. S8(b): Snapshot of single fluorescence beads (incident angle: 68°). The same tendency was confirmed at the incident angle of 68° (TIR) as well. The maximum fluorescence intensity was observed on AuOA sheet covered with 20 nm SiO_2 layer. Although the FWHM values were the same for all the images, the foot section of the profile (the width at 10% of the maximum intensity) was narrower on AuOA sheet compared with that on glass. The wavelength and the intensity of the incident laser was the same as **Fig. S8(a)**. The exposure time was 100 msec for all images.

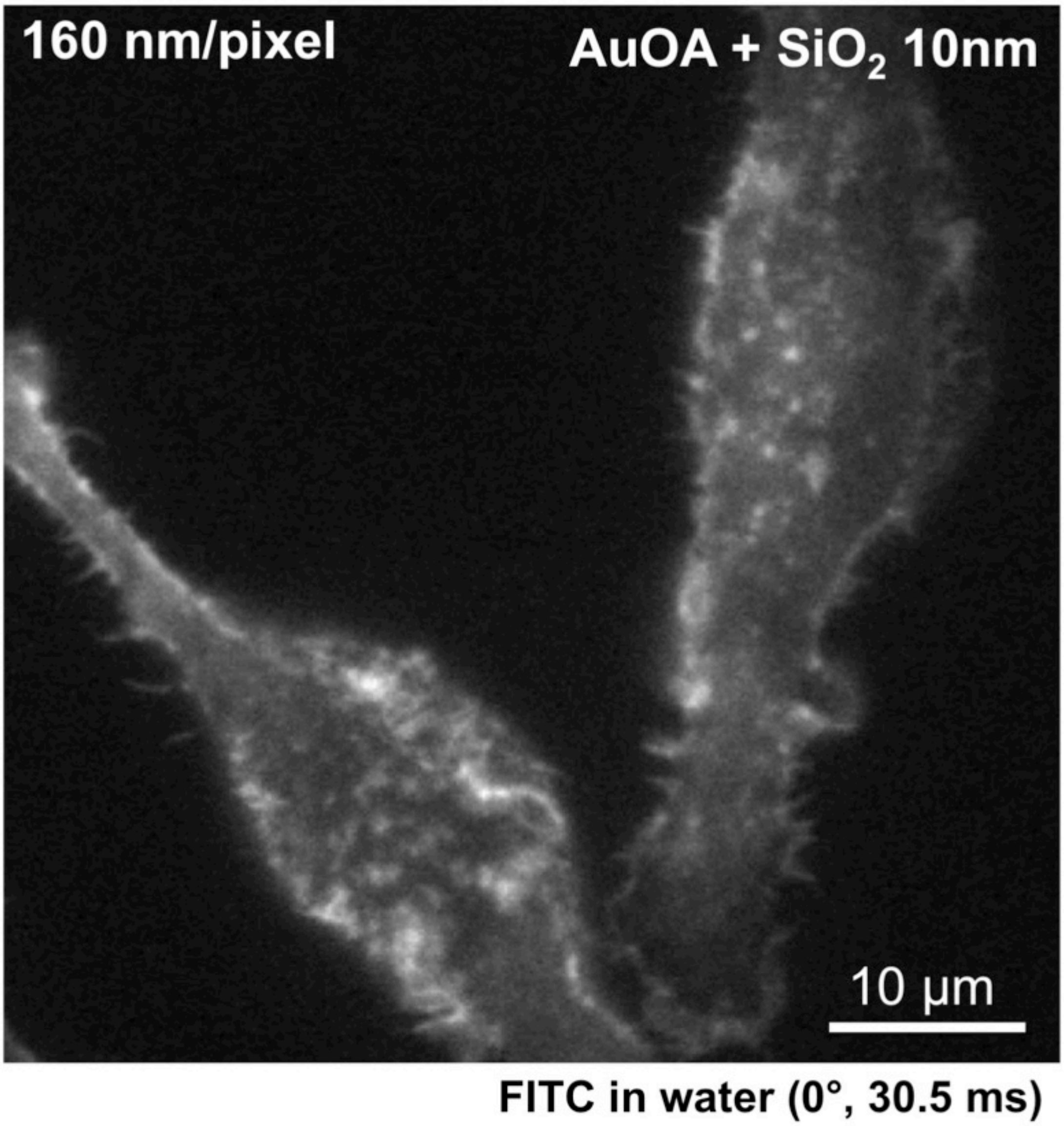

Supplementary Fig. S9: Fluorescence image of FITC-labeled RBL-2H3 on the SiO_2-AuOA sheet taken with a regular TIRF-CCD camera. The image was taken under an epifluorescence microscope (incident angle: 0°) in aqueous medium with a regular TIRF microscope CCD camera (160 nm/pixel) for comparison with that taken by the super-resolution digital CMOS camera (65 nm/pixel) shown in **Fig. 5**.

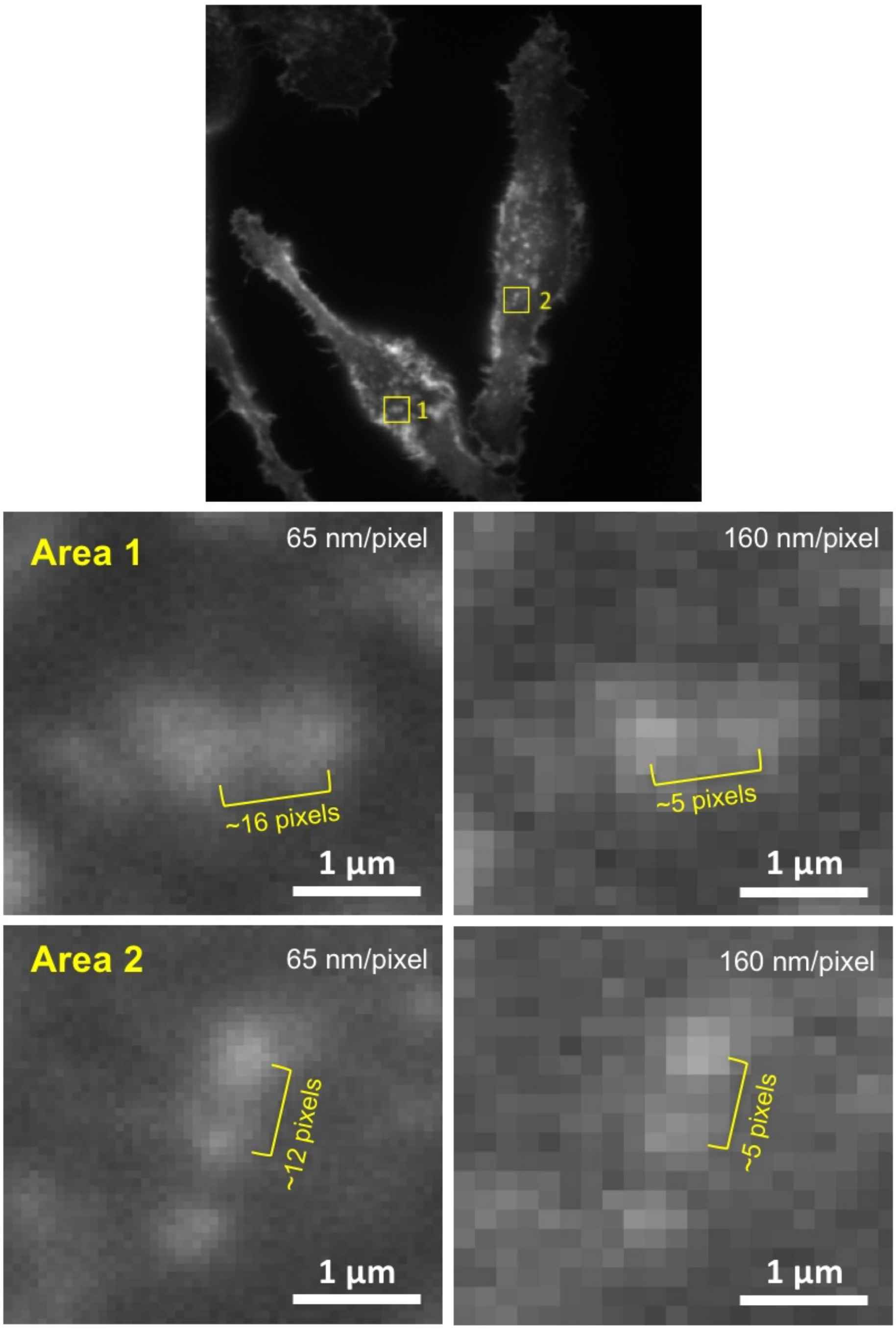

Supplementary Fig. S10: Magnified images of Fig. 5 and Supplementary Fig. S9. The magnified images from the digital CMOS camera (65 nm/pixel, Fig. 5) and CCD camera (160 nm/pixel, Fig. S9) for the two selected areas were compared to confirm the lateral resolution of the cell imaging on the AuOA sheet. The image can resolve the bright spots separated by approximately 1 µm, but the digital CMOS camera allowed for a short distance to be determined. The quantitative comparison of the lateral resolution between the image on the glass and AuOA is shown in **Supplementary Fig. S8**.

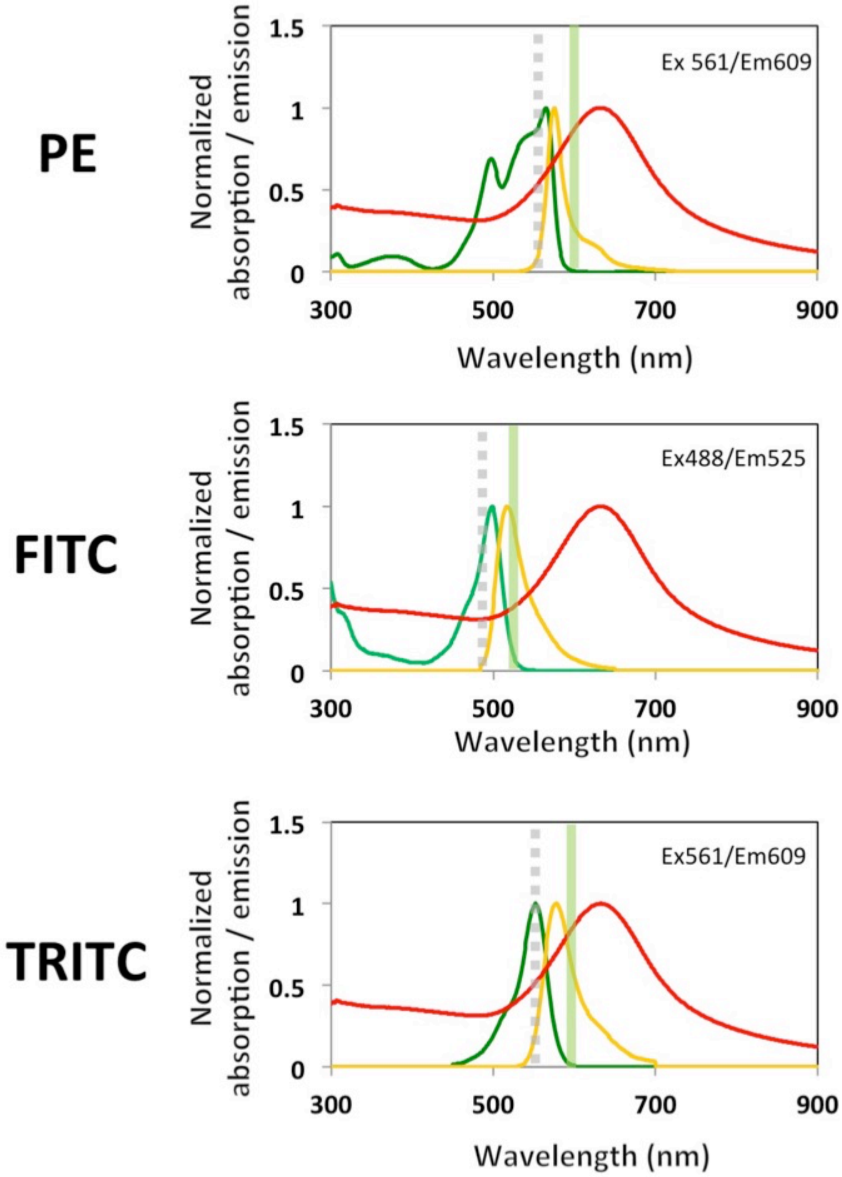

Supplementary Fig. S11: Spectrum overlap between the LSPR band of the AuOA sheet and the fluorescence spectra of the fluorescent dyes. The red line corresponds to the LSPR band of the AuOA sheet, and the green and yellow lines correspond to the excitation/emission spectra, respectively, of PE, FITC and TRITC dyes. The dashed gray and light green lines indicate the wavelengths of the excitation lasers and emission filters, respectively.

Supplementary Video S1: Live cell imaging of Venus-paxillin-expressing NIH-3T3 cells on an AuOA sheet.

Dynamic live cell image of Venus-paxillin-expressing NIH-3T3 cells on an AuOA sheet covered with a 10-nm-thick SiO_2 layer under a TIRF microscope (exposure time: 500 msec, duration: 140 sec). The cells were trypsinized, seeded onto the sheet and maintained in the CO_2 incubator overnight prior to imaging. The imaging was performed in a humidified temperature-controlled chamber at 37°C. The laser intensity was 1 mW. The excitation wavelength of the laser was 514 nm (542 nm emission filter), and the incident angle was 75° (TIR). The video images were obtained as a 50-tuple speed without image processing (**A**) and with threshold control (**B**). The frame size is 2048 x 2048 pixels (133 μm x133 μm). The short-term dynamics of each focal adhesion were clearly visualized on an AuOA sheet, which enabled the observation of the behaviors of the pseudopodium and cell shape. Movement of the cell body (gray color) in a short time scale could also be monitored via Venus-paxillin, which was distributed in the cytosol. Because the focal depth is very thin on the AuOA sheet, differences in the degree of focusing for focal adhesions in the entire cell were observed among the three cells. The right and left cells might show a certain short distance between the paxillin position and the AuOA sheet surface. Paxillin is known to be located approximately 30 nm from the plasma membrane[3]. A very small shift of the cell membrane in the Z direction should affect the visualization level of focal adhesions.

Supplementary Video S2: Live cell imaging of Venus-paxillin-expressing NIH-3T3 cells on glass.

Dynamic live cell image of Venus-paxillin-expressing NIH-3T3 cells on a control glass under a TIRF microscope (exposure time: 500 msec, duration: 140 sec). All other conditions were the same as in Video 1. The video images are presented at a 50-tuple speed without image processing (**A**) and with threshold control (**B**). The frame size is 2048 x 2048 pixels (133 μm x133 μm). Because the imaging depth was greater (~100 nm) on glass than on the AuOA sheet, focal adhesions in several cells were visualized in a single focusing plane. Instead, a higher background brightness was observed compared with the observation on the AuOA sheet (Suppl. Video S1), which interferes with the precise characterization of the rapid dynamics of molecular events in each focal adhesion.

Supplementary Video S3: Time-lapse imaging of Venus-paxillin-expressing NIH-3T3 cells on an AuOA sheet.

Time-lapse image of Venus-paxillin-expressing NIH-3T3 cells on an AuOA sheet covered with a 10-nm-thick SiO_2 layer. The exposure time was 10 sec, and the interval time was 10 sec. The incident angle was 75° in (**A**) and 0° in (**B**). All other conditions were the same as in Video S1. The video images are presented at a speed of 3 frames/sec without image processing. Because the exposure time was long (10 sec), the binding sites were displayed as an elongated feature. The frame size is 2048 x 2048 pixels (133 μm x133 μm).

REFERENCES

1. Yoshida, A., Imazu, K., Li, X. H., Okamoto, K. & Tamada, K. Spectroscopic Properties of Multi layered Gold Nanoparticle 2D Sheets. *Langmuir* **28**, 17153-17158 (2012).
2. Toma, M., et al, Collective plasmon modes excited on a silver nanoparticle 2D crystalline sheet, *Phys. Chem. Chem. Phys.* **13**, 7459-7466 (2011).
3. Kanchanawong, P et al. Nanoscale architecture of integrin-based cell adhesions, *Nature* **468**, 580-584 (2010).
